# 

**Published:** 1898-09

**Authors:** 


SEPTEMBER, 1898.

*Vol. XVI.* *No.* **61.**

397945
OCT 21 1898
LIBRARY OF CONGRESS
SMITHSONIAN DEPOSIT.

### THE BRISTOL MEDICO-CHIRURGICAL JOURNAL

**A Quarterly Journal of the Medical Sciences for the West of England and South Wales**

*PUBLISHED UNDER THE AUSPICES OF*
*THE BRISTOL MEDICO-CHIRURGICAL SOCIETY.*

"Scire est nescire, nisi id me

Scire alius sciret."

*Editor:* R. SHINGLETON SMITH, M.D., B.Sc.

WITH WHOM ARE ASSOCIATED

J. MICHELL CLARKE, M.A., M.D., F. R. CROSS, M.B.,
W. H. HARSANT, AND JAMES SWAIN, M.S., M.D.

*Assistant-Editor:* L. M. GRIFFITHS.

*Editorial Secretary:* B. M. H. ROGERS, B.A., M.D., B.CH.

BRISTOL: J. W. ARROWSMITH
LONDON: J. & A. CHURCHILL, 7 GREAT MARLBOROUGH STREET.

*Price One Shilling and Sixpence.*

